# Propionic and Methylmalonic Acidemias: Initial Clinical and Biochemical Presentation

**DOI:** 10.1155/2020/7653716

**Published:** 2020-11-21

**Authors:** Amira Mobarak, Heba Dawoud, Wesam A. Mokhtar, Abdelrahim A. Sadek, Gihan Mohamed Bebars, Amr Ahmed Othman, Rofaida M. Magdy, Hanaa Nofal, Amr Zoair

**Affiliations:** ^1^Medical Biochemical Diseases Division, Pediatrics Department, Faculty of Medicine, Tanta University, Egypt; ^2^Pediatrics Department, Faculty of Medicine, Zagazig University, Egypt; ^3^Neurology Division, Pediatrics Department, Faculty of Medicine, Sohag University, Egypt; ^4^Pediatrics Department, Minia University, Egypt; ^5^Medical Genetics Division, Pediatrics Department, Faculty of Medicine, Sohag University, Egypt; ^6^Clinical Pathology Department, Faculty of Medicine, Tanta University, Egypt; ^7^Pediatrics Department, Faculty of Medicine, Tanta University, Egypt

## Abstract

PA and MAA have numerous nonspecific presentations, potentially leading to delayed diagnosis or misdiagnosis. In this paper, we present the clinical and biochemical characteristics of MMA and PA patients at initial presentation. *Results*. This is a retrospective review of 20 patients with PA (*n* = 10) and MMA (*n* = 10). The most observed symptoms were vomiting (85%) and refusing feeding (70%). Ammonia was 108.75 ± 9.3 *μ*mol/l, showing a negative correlation with pH and bicarbonate and positive correlation with lactate and anion gap. Peak ammonia did not correlate with age of onset (*r* = 0.11 and *p* = 0.64) or age at diagnosis (*r* = 0.39 and *p* = 0.089), nor did pH (*r* = 0.01, *p* = 0.96; *r* = −0.25, *p* = 0.28) or bicarbonate (*r* = 0.07, *p* = 0.76; *r* = −0.22, *p* = 0.34). There was no correlation between ammonia and C3 : C2 (*r* = 0.1 and *p* = 0.96) or C3 (*r* = 0.23 and *p* = 0.32). The glycine was 386 ± 167.1 *μ*mol/l, and it was higher in PA (*p* = 0.003). There was a positive correlation between glycine and both pH (*r* = 0.56 and *p* = 0.01) and HCO_3_ (*r* = 0.49 and *p* = 0.026). There was no correlation between glycine and ammonia (*r* = −0.435 and *p* = 0.055) or lactate (*r* = 0.32 and *p* = 0.160). *Conclusion*. Clinical presentation of PA and MMA is nonspecific, though vomiting and refusing feeding are potential markers of decompensation. Blood gas, lactate, and ammonia levels are also good predictors of decompensation, though increasing levels of glycine may not indicate metabolic instability.

## 1. Introduction

Propionic acidemia (PA) and methylmalonic acidemia (MMA) are autosomal recessive diseases. They are caused by a defect in propionyl-CoA carboxylase and methylmalonyl-CoA enzymes, respectively.

They present classically in an “early-onset” neonatal acute life-threatening form, including lethargy, vomiting, shock, dehydration, metabolic acidosis, and hyperammonemia. This acute presentation is known as “acute decompensation” which can be fatal if not treated properly. PA and MMA can also present later in life with chronic and more insidious onset. While more common in early onset, both suffer from acute decompensation episodes throughout the course of the illness. These episodes are usually triggered by infection, fasting, fever, or any other stress condition [1, 2].

A wide array of presenting symptoms, which are almost always nonspecific, can lead to delayed diagnosis or misdiagnosis. Multiple reports have addressed the primary presentation of patients with PA and MMA, each describing different symptoms overall as well as different most frequent symptoms [3–11], including atypical neurological presentation [12–14].

This paper reports the clinical and biochemical characteristics of 20 Egyptian patients with MMA and PA at first presentation, with the hopes of enhancing knowledge surrounding the presenting signs and symptoms of these two conditions.

## 2. Patients and Methods

We reviewed 20 Egyptian patients with PA (*n* = 10) and MMA (*n* = 10) diagnosed from year 2014 to 2018. This study was approved by the ethics committee of the Faculty of Medicine, Tanta University, Egypt. Written informed consent was obtained from the patients' legal guardians/parents. All data was collected retrospectively from patient charts.

### 2.1. Statistical Analysis

The data collected and inserted into a Microsoft Excel spreadsheet for analysis are presented in the form of mean ± standarddeviation (SD). The Shapiro-Wilk test was used to check for data normality. Mann-Whitney, Student's *t*-test, and Fisher's exact tests were used to compare data groups. Pearson's linear correlation was used to detect the relationship between different variables. A multiple regression analysis was calculated to predict the ammonia level of the cohort based on the lactate and bicarbonate levels. Significance value was set at *p* < 0.05.

## 3. Results

### 3.1. Clinical Presentation

This study included 20 patients, PA (*n* = 10) and MMA (*n* = 10) with age 3.99 ± 1.29 years at the time of data collection. Patients were diagnosed by selective screening prompted by positive family history (one patient) or suspicious preliminary investigations and clinical presentation. All patients were of a Middle Eastern background and born at term (*m* = 38.6 ± 2.18); 80% had a history of consanguinity, and 45% were females. The majority of patients (70%) were delivered by spontaneous vaginal delivery. The birth weight was 3.9 ± 1.2 kg, with no significant difference between PA (3.5 ± 1.33 kg) and MMA (4.4 ± 1.17 kg) (*p* = 0.16; two tail).

The age of onset of symptoms was 5.38 ± 1.38 months, with 60% of patients presenting in the first 3 months of life, only three (15%) in the neonatal period, and 40% after 3 months of age with no significant difference between PA (4.35 ± 6.29 months) and MMA (6.32 ± 6.25 months), and *p* = 0.49. The age at diagnosis was 8.05 ± 7.6 months, with no significant difference between MMA (8.7 ± 6.8 months) and PA (8.05 ± 8.8 months) (*p* = 0.85; two tail). The time to diagnosis after the onset of initial symptoms was 3.03 ± 5.5 months, with no difference between PA (3.7 ± 7.6 months) and MMA (2.37 ± 2.26 months) (*p* = 0.6); two tail), or between early onset (3.8 ± 6.8 months) and late onset (1.77 ± 2.5months) (*p* = 0.14; two tail).

Almost all patients, regardless of age at diagnosis, presented with acute metabolic decompensation apart from one patient who presented with acute ataxia without signs of metabolic derangement. The most frequent presenting symptoms were vomiting (85%) followed by refusing feeding (70%), dehydration (60%), encephalopathy (55%), hypotonia (55%), shock (45%), hepatomegaly (45%), seizures (20%), dystonia (10%), and ataxia (5%). There was no significant statistical difference between PA and MMA in frequency of presenting symptoms, (*p* > 0.05) except for hypotonia which was more frequent in PA (*p* = 0.038; two tail). The triggering factors were fever and GI illness in 45% of the patients and upper respiratory infection in 40%, and no trigger factor could be identified in 15%.

### 3.2. Biochemical Presentation

The pH was 7.32 ± 0.06. The standard bicarbonate was 12.8 ± 5.71 mmol/l. Severe metabolic acidosis with bicarbonate < 10 mmol/l was found in 35% (7/20); 45% (9/20) had bicarbonate 10-16 mmol/l, and 20% (4/20) had bicarbonate > 16 mmol/l. The anion gap was 22.5 ± 8.38, and the MMA patients tended to have higher anion gap, with *p* = 0.005 (Tables 1 and 2). Nineteen patients (19/20) had positive ketones in their urine with a median of +2, with no significant difference between the PA and MMA groups (*p* = 0.53).

Peak ammonia level at presentation was 108.75 ± 9.3 *μ*mol/l (ref 9: 33 *μ*mol/l), with 10 patients having an ammonia > 100 *μ*mol/l. Peak ammonia level did not show any correlation with age of onset of symptoms (*r* = 0.11 and *p* = 0.64) or age at diagnosis (*r* = 0.39 and *p* = 0.089), nor did the pH (*r* = 0.01, *p* = 0.96; *r* = −0.25, *p* = 0.28) or bicarbonate levels (*r* = 0.07, *p* = 0.76; *r* = −0.22, *p* = 0.34), respectively. There was also no significant correlation between the ammonia level and C3 : C2 (*r* = 0.1 and *p* = 0.96) or C3 (*r* = 0.23 and *p* = 0.32).

**Figure 1:**
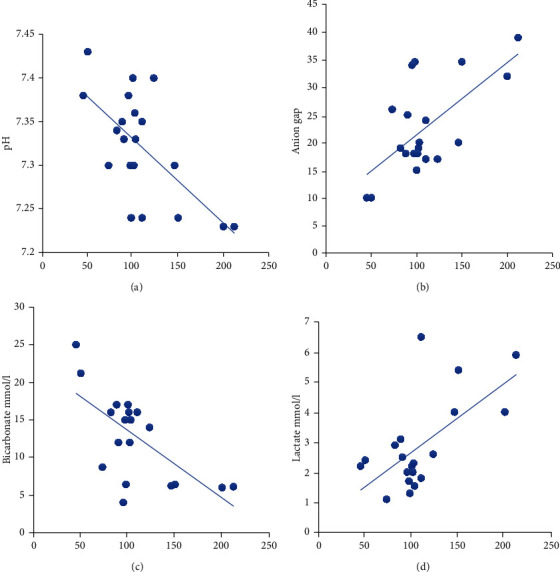
Correlation between the ammonia in *μ*mol/l (*x*-axis) and other biochemical markers.

Ammonia level, however, had significant negative correlations with both pH and bicarbonate levels and a positive correlation with lactate and anion gap (Figure 1). A multiple regression analysis was calculated to predict the ammonia level of the cohort based on the lactate and bicarbonate levels. The results of the regression suggest that level of lactate and bicarbonate can predict 67% of the variance (*r*^2^ = 0.67, *F*(2.17) = 16.99, and *p* < 0.001). It was found that lactate significantly predicted ammonia level (*β* = 0.52 and *p* = 0.004), as did bicarbonate (*β* = −0.47 and *p* = 0.008), at a confidence interval of 95%.

Lactate level was 2.87 ± 1.53 mmol/l, where (11/20) patients had lactate above 2.2 mmol/l. Interestingly, lactate did not show significant correlation with alanine (*r* = −0.3 and *p* = 0.14).

**Figure 2:**
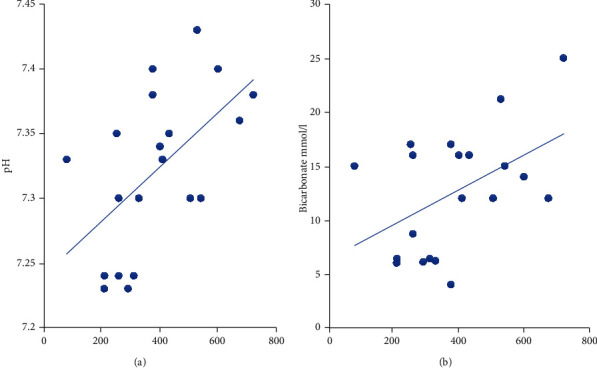
Correlation between the glycine in *μ*mol/l on *x*-axis and (a) pH and (b) the standard bicarbonate levels.

Glycine level was 386 ± 167.1 *μ*mol/l and tended to be significantly higher in PA (*p* = 0.003). There was a positive correlation between glycine and both pH (*r* = 0.56 and *p* = 0.01) and HCO_3_ (*r* = 0.49 and *p* = 0.026). No significant correlation was found between glycine and both ammonia (*r* = −0.435 and *p* = 0.055) and lactate (*r* = 0.32 and *p* = 0.160) (Figure 2).

MMA patient age at diagnosis was 8.7 ± 6.8 months, with all patients presenting after one month of life. The MMA group had a C3 : C2 of 0.75 ± 0.52 and a C3 of 14.3 ± 5.36 *μ*mol/l. PA patient age at diagnosis was 8.05 ± 2.7 months, with a C3 value of 17.6 ± 3.99 *μ*mol/l and C3 : C2 of 1.11 ± 0.14. Although C3 was higher in the PA group, it was not statistically significant (*p* = 0.14), and the C3 : C2 ratio did not show any significant difference between both groups (*p* = 0.06; two tail).

The mean level of uric acid was 4.45 ± 1.85 mg/dl (264.7 ± 110.04 *μ*mol/l). One MMA patient had uric acid of 11.2 mg/dl (666.23 *μ*mol/l) and was on allopurinol for four days. Mean level of random blood glucose (RBG) was 156.6 ± 44.2 mg/dl (8.7 ± 2.47 mmol/l); in 15/20 (75%), it was >126 mg/dl (7 mmol/l) (Table 3).

Mean albumin level was 35.8 ± 4.7 gm/l. It tended to be lower in PA (33.3 ± 4.8 g/l) when compared to MMA (38.3 ± 3.3 g/l and *p* = 0.014). Five patients had a whitebloodcellcount ≤ 3,500 cells/mm^3^, and one patient had platelets of 157,000/mm^3^. Six patients had hemoglobin of <10.5 g/dl.

## 4. Discussion

The study is aimed at describing the clinical and biochemical presentations of MMA (*n* = 10) and PA (*n* = 10) in an Egyptian cohort of patients. All patients in this study were born at full term (GA > 37 weeks) or decrease in birth weight. This is in contrast with Kolker et al. [6] who noted that patients with MMA tended to have low birth weight, hence postulating that there could be a potential intrauterine disease process in the form of intrauterine impaired energy metabolism. Interestingly, PA patients in their cohort did not have low birth weight.

In our cohort, most patients (55%) presented in the first 3 months of life and only 15% presented in the neonatal period. This is in contrast to previous studies that demonstrated a higher frequency of patients presenting in the neonatal period [3, 5, 6, 10]. Sass et al. reported that 92% of their patients presented in the first 90 days of life, with 74% of their early-onset group presenting within the first 8 days of life and 26% between 11 and 90 days [9]. It was previously reported that patients with late onset tended to have a more obvious diagnostic time delay [6]. However, there was no correlation between the diagnostic time delay and age of onset in our cohort.

Previous studies show patients with late onset commonly presenting with chronic neurological symptoms, developmental delay, or organ damage rather than acute metabolic decompensation (which was reported to be more common in early onset) [6, 10]. However, all patients in our cohort presented with metabolic decompensation of different severities regardless of age of onset, except one patient who presented with acute ataxia without any signs of metabolic decompensation. In addition, the neurological symptoms of seizures and dystonia were in the context of an episode of metabolic decompensation.

We must acknowledge that this is a retrospective study, and the age of onset of symptoms was concluded based on medical records and history taking. It is possible that earlier, more subtle symptoms, which may have in fact been more accurate indicators of age of onset, were not recorded through these means; obviously only major events, such as admissions and ER visits, can be reliably captured, while nonmajor events, which may still cause significant clinical burden, are more difficult to ascertain. In addition, genotype is one of the factors that can help establish severity, a factor that is unfortunately unavailable for our cohort.

The most commonly observed initial symptoms in our cohort were vomiting and refusing feeding, indicating that these are major clinical signs [15, 16] and should alert the physician of possible decompensation. We did not note any significant difference between PA and MMA patients with regard to the frequency of presenting symptoms, with the exception of hypotonia which tended to be more frequent in PA. Hypotonia was described previously as a frequent neurological complication of PA whether at initial presentation or later on [2].

We noted that ammonia had a negative correlation with pH and bicarbonate levels and a positive correlation with the anion gap and lactate. These biochemical abnormalities reflect secondary metabolic changes induced by accumulating toxic metabolites/organic acids including propionic acid. These metabolites inhibit PDH and TCA cycle enzymes and decrease succinyl-CoA production. In addition, they inhibit CPS I enzyme either directly or indirectly through N-acetyl glutamate synthase inhibition [17, 18]. We also observed that bicarbonate and lactate can be good predictors of hyperammonemia. By using these three markers together works to determine a more specific decompensation state of the patient [15, 16].

Unlike [6] who noted that late-onset patients had more severe metabolic acidosis compared to early-onset patients, with early-onset patients having higher ammonia levels, in our cohort, ammonia and pH did not correlate with age of onset or age at diagnosis. Interestingly, we found a positive correlation between glycine level and both pH and bicarbonate level, aligning with the hypothesis postulated by Al-Hassnan et al. [19]. They postulated that the conjugation of propionate with glycine or carnitine for excretion is pH sensitive, and, at normal acid-base status, propionyl carnitine might be the preferred mechanism; this is in contrast with metabolic acidosis when propionyl glycine might be the predominant form of propionate excretion.

## 5. Conclusion

Clinical presentation of PA and MMA can vary, presenting a hurdle for timely diagnosis and management. For any patient who presents with nonspecific symptoms in the context of a minor metabolic stress, a metabolic disorder should be suspected. Vomiting and refusing feeding are also “red flags” to potential decompensation. In addition, our data indicates that blood gas, lactate, and ammonia levels are good predictors of acute decompensation when used in combination with patients' biochemical evaluation. Increasing levels of glycine might not be an indicator of metabolic instability in such patients.

## Figures and Tables

**Table 1:** Laboratory parameters at initial presentation.

Lab. parameter	Range (SI)	Mean ± SD	Normal reference range
pH	7.23-7.43	7.32 ± 0.06	7.35-7.45
HCO_3_	4-25	12.8 ± 5.71 mmol/l	22-26
Anion gap	10-39	22.5 ± 8.38	12-15
Ammonia	45-212	108.75 ± 41.82 *μ*mol/l	9-33 *μ*mol/l
Lactate	1.1-6.5	2.87 ± 1.53 mmol/l	1-2.2 mmol/l
Blood glucose	67-250 (3.7-13.8 mmol/l)	156.65 ± 44.21 mg/dl (8.7 ± 2.47 mmol/l)	70-126 mg/dl
Uric acid	2.5-11.2 (148.71-666.23 *μ*mol/l)	4.45 ± 1.85 mg/dl (264.7 ± 110.04 *μ*mol/l)	3.5-4.5 mg/dl
Glycine	77.5-721	386.56 ± 167.11 *μ*mol/l	20-500 *μ*mol/l
Alanine	99.56-401	245.97 ± 88.65 *μ*mol/l	20-600 *μ*mol/l
C3	9.98-27.7	16.06 ± 4.89 *μ*mol/l	0-7 *μ*mol/l
C3 : C2	0.31-1.68	0.93 ± 0.42 *μ*mol/l	0-0.25 *μ*mol/l
Urea	12-50 (1.99-8.3 mmol/l)	25.9 ± 8.36 mg/dl (4.31 ± 1.39 mmol/l)	10-45 mg/dl
Creatinine	0.4-0.8 (35.37-70.74 *μ*mol/l)	0.62 ± 0.12 mg/dl (54.82 ± 10.61 *μ*mol/l)	0.6-1 mg/dl
AST	17-74	41.45 ± 14.06 U/l	20-45
ALT	20-69	37.7 ± 13.41 U/l	20-45
Albumin	29-45	35.8 ± 4.8 g/l	35-50 g/dl
Hemoglobin	87-120	107 ± 10.4 g/l	100 − 115 g/l
Platelets	157,000-470,000	327,950 ± 101,710	150 − 450 × 10^3/^mm^3^
White blood cells	2700-7000	4490 ± 1121.98	3,500-5000/mm^3^

**Table 2:** Comparison between the initial laboratory parameters of PA and MMA patients.

Lab parameter	PA	MMA	*p* value
	Mean ± SD (SI)		
pH	7.34 ± 0.05	7.3 ± 0.06	0.14
HCO_3_ (mmol/l)	14.84 ± 5.8	10.76 ± 4.97	0.11
Anion gap	17.6 ± 6.13	27.42 ± 7.5	0.005^∗^
Ammonia (*μ*mol/l)	107.4 ± 44.39	110.1 ± 41.44	0.70
Lactate	2.52 ± 0.82	3.22 ± 1.99	0.70
Blood glucose (mg/dl)	154.7 ± 47.12 (8.5 ± 2.5 mmol/l)	158.6 ± 43.55 (8.8 ± 2.4)	0.84
Uric acid (mg/dl)	4.01 ± 0.56 (238.5 ± 33.31 *μ*mol/l)	4.88 ± 2.55 (290.28 ± 151.68 *μ*mol/l)	0.81
Glycine (*μ*mol/l)	490.26 ± 158.8	282.84 ± 99.08	0.0032^∗^
Alanine (*μ*mol/l)	243.11 ± 84.42	248.83 ± 97.19	0.88
C3 (*μ*mol/l)	17.69 ± 3.94	14.43 ± 5.36	0.14
C3 : C2	1.11 ± 0.14	0.75 ± 0.52	0.06
Urea (mg/dl)	28.5 ± 11.21 (4.74 ± 1.86 mmol/l)	23.3 ± 2.58 (3.87 ± 0.47)	0.10
Creatinine (mg/dl)	0.65 ± 0.09 (57.46 ± 7.95 *μ*mol/l)	0.58 ± 0.13 (51.27 ± 11.49 *μ*mol/l)	0.36
AST (U/l)	47.8 ± 16.41	47.8 ± 35.1	0.04^∗^
ALT (U/l)	40.2 ± 17.67	35.2 ± 7.28	0.42
Albumin (g/l)	33.3 ± 4.8	38.3 ± 3.2	0.01^∗^
Hemoglobin (g/l)	114.3 ± 16.3	105.7 ± 10.9	0.22
White blood cells (cells/mm^3^)	4280 ± 1444.37	4700 ± 687.99	0.42
Platelets × 10^3^/mm^3^	330.4 ± 105.12	325.5 ± 103.79	0.91

^∗^Significance value *p* < 0.05.

**Table 3:** Blood glucose level.

Blood glucose level	Number of patients
67 mg/dl	1
70-140 mg/dl	5
140-180 mg/dl	11
180-200 mg/dl	1
>200 mg/dl	2

## Data Availability

The datasets generated and/or analyzed during the current study are available from the corresponding author upon reasonable request.

## Additional Points

*Take Home Message*. PA and MMA have nonspecific presenting symptoms, though vomiting and refusing feeding are “red flags” for decompensation. Moreover, blood gas and lactate taken with ammonia level are also good markers for biochemical instability.
